# Impact of pegfilgrastim approval on relative dose intensity and outcomes of R-CHOP for diffuse large B-cell lymphoma

**DOI:** 10.1097/MD.0000000000029028

**Published:** 2022-03-11

**Authors:** Yuka Morita, Yusuke Kanemasa, Yuki Sasaki, An Ohigashi, Taichi Tamura, Shohei Nakamura, Yu Yagi, Akihiko Kageyama, Yasushi Omuro, Tatsu Shimoyama

**Affiliations:** a Department of Medical Oncology, Tokyo Metropolitan Cancer and Infectious Diseases Center, Komagome Hospital, Tokyo, Japan; b Department of Clinical Research Support, Tokyo Metropolitan Cancer and Infectious Diseases Center, Komagome Hospital, Tokyo, Japan.

**Keywords:** diffuse large B-cell lymphoma, febrile neutropenia, pegfilgrastim, relative dose intensity, survival

## Abstract

Maintaining relative dose intensity (RDI) of rituximab, cyclophosphamide, doxorubicin, vincristine, and prednisone (R-CHOP) improves the prognosis of patients with diffuse large B-cell lymphoma (DLBCL). Pegfilgrastim was approved in Japan in November 2014 to prevent febrile neutropenia (FN) and maintain RDI.

In this retrospective study, we reviewed 334 patients with DLBCL who received 6 or more courses of R-CHOP and analyzed the differences in the RDI, overall survival (OS), and progression-free survival between patients whose treatment started after November 2014 (postapproval group) and those whose treatment started before October 2014 (pre-approval group).

The incidence of FN was lower (20% vs 38.3%, *P* < .001) and the RDI of R-CHOP was higher (86.8% vs 67.8%, *P* < .001) in the postapproval group. Pegfilgrastim was administered to many of these patients (76.8%) and was thought to have contributed to the high RDI maintenance in the postapproval group. Interrupted time-series analysis showed a significant rise of the RDI at the timing of pegfilgrastim approval in patients aged <70 years (estimated change: 18.1%, *P* < .001). The 5-year OS (85.7% vs 69.9%, *P* = .009) and progression-free survival (81.4% vs 64.4%, *P* = .011) were superior in the postapproval group. However, the differences were not significant in matched-pair analysis matching National Comprehensive Cancer Network-International Prognostic Index scores. Improved survival outcomes in this group were observed only among patients with Ann Arbor stage 3/4 (5-year OS: 83.7% vs 61.3%, *P* = .019) and high-risk on the National Comprehensive Cancer Network-International Prognostic Index (5-year OS: 80.7% vs 32.4%, *P* = .014). Multivariate analysis showed that a high RDI and low lactate dehydrogenase were associated with superior OS (RDI ≥ 85%, hazard ratio: 0.48, *P* = .016; lactate dehydrogenase > institutional upper limit of normal, hazard ratio: 2.38, *P* = .005).

The RDI of R-CHOP was able to be maintained at higher levels, the incidence of FN was lower, and significantly better clinical outcomes were achieved in clinically high-risk groups after pegfilgrastim approval. Maintaining a high RDI in R-CHOP by administering pegfilgrastim to those who are likely to have low RDI without it is important for achieving favorable outcomes in patients with DLBCL.

## 1 Introduction

Diffuse large B-cell lymphoma (DLBCL) is the most common subtype of non-Hodgkin lymphoma (NHL) and accounts for approximately 30% to 40% of NHL cases. Standard therapy for DLBCL is combination chemotherapy with rituximab, cyclophosphamide, doxorubicin, vincristine, and prednisone (R-CHOP). Several retrospective studies have reported that maintaining the relative dose intensity (RDI) of R-CHOP significantly improved clinical outcomes in patients with DLBCL, and that the RDI was an independent factor associated with response and survival prognosis.^[1–3]^ Although DLBCL is a potentially curable disease, approximately one-third of patients will eventually experience a relapse, and the prognosis is extremely poor for those who relapse after front-line therapy.^[4]^

Pegfilgrastim, a long-acting granulocyte-colony stimulating factor (G-CSF), was approved for use in Japan in November 2014 and has been administered to patients with various types of malignant tumor. Several studies have shown that prophylaxis with G-CSFs reduces the incidence of febrile neutropenia (FN) and mortality and increases the RDI, and that pegfilgrastim is more effective than short-acting G-CSFs, such as filgrastim or lenograstim.^[5–8]^ Pegfilgrastim is used in clinical practice not only to prevent chemotherapy-induced FN but also to maintain the RDI of chemotherapy, especially in elderly patients, and is thought to enable a higher RDI necessary for achieving better clinical outcomes. However, it is unclear whether pegfilgrastim actually contributes to better survival outcomes in patients with DLBCL.

The present retrospective study evaluated the effect of pegfilgrastim on the RDI of R-CHOP and clinical outcomes in patients with DLBCL by comparing the clinical data before and after approval of the drug.

## 2 Methods

### 2.1 Study design and patients

The medical records of patients in whom DLBCL was newly diagnosed between August 2004 and March 2018 at our hospital were reviewed. Data on patients who received more than 6 courses of R-CHOP with or without radiotherapy or prevention of central nervous system relapse were analyzed. Patients who received other regimens, and those with HIV-associated lymphoma, and primary central nervous system lymphoma were excluded. The study population was divided into those who started receiving chemotherapy after pegfilgrastim approval in November 2014 (the postapproval group) and those who began receiving chemotherapy before October 2014 (the pre-approval group).

DLBCL was pathologically diagnosed in accordance with the World Health Organization classification,^[9,10]^ and clinical staging was performed using the Ann Arbor classification. Performance status (PS) was evaluated using the Eastern Cooperative Oncology Group (ECOG) criteria. The National Comprehensive Cancer Network-International Prognostic Index (NCCN-IPI) scores were calculated based on age, serum lactate dehydrogenase (LDH), PS, Ann Arbor stage, and extranodal involvement at diagnosis.^[11]^

This retrospective study was conducted in accordance with the Declaration of Helsinki and approved by the Ethics Committee of Tokyo Metropolitan Cancer and Infectious Diseases Center, Komagome Hospital. Written informed consent was waived because this study used retrospective data obtained from the hospital medical records.

### 2.2 Treatment

Standard R-CHOP therapy consisting of rituximab (375 mg/m^2^ on day 1), cyclophosphamide (750 mg/m^2^ on day 2), doxorubicin (50 mg/m^2^ on day 2), vincristine (1.4 mg/m^2^ [maximum 2 mg/body] on day 2), and prednisone (100 mg/day on days 2–6) was administered every 3 weeks. The dosage was often reduced to 5/6 in patients aged 70 to 79 years and to 7/12 in patients aged over 80 years in accordance with a previous report.^[12]^ The dosage, timing of the start of subsequent cycles, and preparations for G-CSF administration were determined at the physician's discretion.

### 2.3 Outcome measures

The delivered dose intensity was calculated as the total delivered dose divided by the total time until completion of the chemotherapy. The RDI was calculated as the percentage of the delivered dose intensity divided by the standard intensity. The average RDI for cyclophosphamide and doxorubicin was used for statistical analysis. Overall survival (OS) was defined as the period from the initiation of chemotherapy to the last follow-up or death from any cause. Progression-free survival (PFS) was defined as the period from the initiation of chemotherapy to progression, relapse, last follow-up, or death from any cause.

### 2.4 Statistical analysis

OS and PFS were estimated using the Kaplan-Meier method and were compared using univariate analysis with the log-rank test. Matched-pair analysis for OS and PFS was performed with 1:1 matching on NCCN-IPI scores. Multivariate analysis was performed for OS using the Cox proportional hazards model. The differences in the characteristics between the 2 groups were assessed by Fisher exact test or Student *t* test. Multivariate analysis of factors associated with RDI ≥85% was performed using logistic regression analysis.

We employed interrupted time-series analysis to examine the trends and change of RDI before and after the pegfilgrastim approval. This analysis is a quasi-experimental design with which to evaluate the longitudinal effects of interventions, through regression modelling.^[13,14]^ We fitted segmented linear regression models using the parameterization of a previous report^[15]^:


$$Y_{t}=\beta_{0}+\beta_{1}\;t+\beta_{2}\;D_{t}+\beta_{3}[t-T_{I}]\;D_{t}+\varepsilon_{t}$$

where *Y*_*t*_ represents the outcome that is measured at time point *t* of N time points (1 to n_1_ measurements during the pre-interruption stage, and n_1_ + 1 to n_2_ measurements in the postinterruption stage), with the interruption occurring at time *T*_*I*_. *D*_*t*_ is an indicator variable that represents the postinterruption interval: coded as 0 in the pre-interruption period, and as 1 in the postinterruption period. The model parameters (*β*_*s*_) represent the baseline intercept (*β*_*0*_); pre-interruption slope (*β*_*1*_); change in level at the interruption (*β*_*2*_), and the change in slope (*β*_*3*_). The error term *ε*_*t*_ allows for deviation from the fitted model.

All *P* values were 2 sided, and *P* < .05 was considered to indicate statistical significance. Statistical analyses were performed with R (The R Foundation for Statistical Computing, Vienna, Austria).

## 3 Results

### 3.1 Patient characteristics

**Figure 1:**
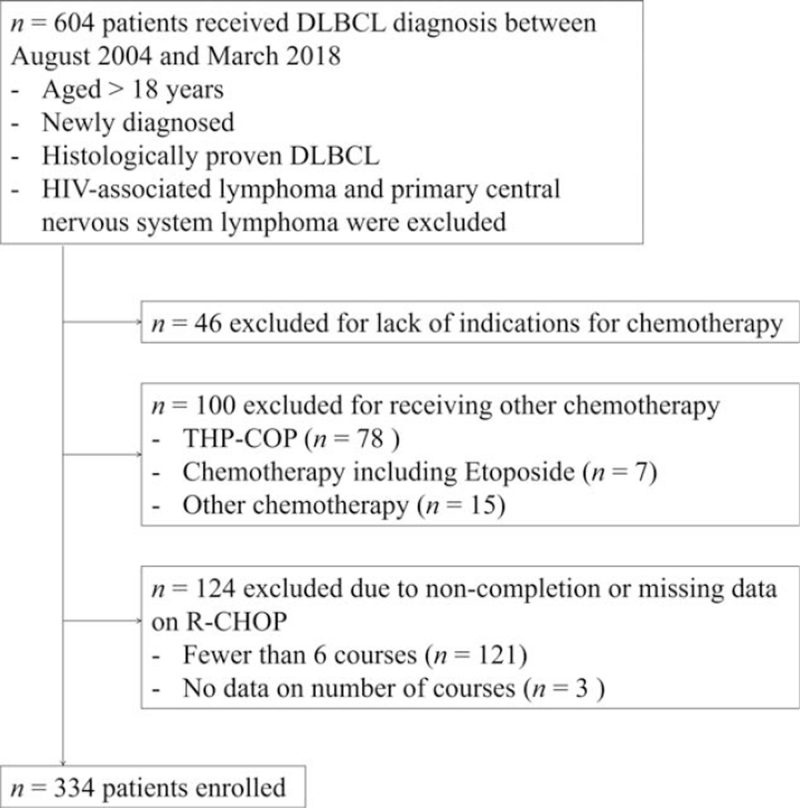
Flow chart of patient selection.

Between August 2004 and March 2018, DLBCL was newly diagnosed in 604 patients. In total, 334 patients met the inclusion criteria and were analyzed (Fig. 1). The median follow-up time for all patients was 59 months (range: 7–192 months) (71 months [range: 7–192 months] for pre-approval group and 40 months [range: 7–67 months] for postapproval group). The patients were classified into the postapproval (n = 125) and pre-approval (n = 209) groups. Table 1 shows the patient characteristics. Patients with Ann Arbor stage 3/4 (46.4% vs 62.2%, *P* = .006), high-intermediate or high risk on the NCCN-IPI (40% vs 57.4%, *P* = .002), and serum albumin <3.7 g/dL (29.6% vs 43.1%, *P* = .015) were fewer in the postapproval group than in the pre-approval group. The 2 groups did not differ significantly in terms of the other factors.

**Table 1:** Patient characteristics.

	Postapproval (n = 125)	Pre-approval (n = 209)	
	n (%)	n (%)	*P* value
Age (>60 yrs)	89 (71.2)	146 (69.9)	.81
Sex (male)	65 (52.0)	126 (60.3)	.17
B-symptoms (+)	31 (24.8)	62 (29.7)	.38
ECOG-PS (≥2)	18 (14.4)	47 (22.5)	.086
LDH (>ULN)	58 (46.4)	120 (57.4)	.055
Ann Arbor stage (3/4)	58 (46.4)	130 (62.2)	.006
Extranodal involvement (≥2)	31 (24.8)	59 (28.2)	.53
NCCN-IPI (HI/H)	50 (40.0)	120 (57.4)	.002
CCI (≥3)	51 (40.8)	66 (31.6)	.098
Serum albumin (<3.7 g/dL)	37 (29.6)	90 (43.1)	.015

CCI = Charlson Comorbidity Index, ECOG-PS = Eastern Cooperative Oncology Group performance status, LDH = lactate dehydrogenase, NCCN-IPI = National Comprehensive Cancer Network-International Prognostic Index, ULN = upper limit of normal.

### 3.2 Relative dose intensity

**Figure 2:**
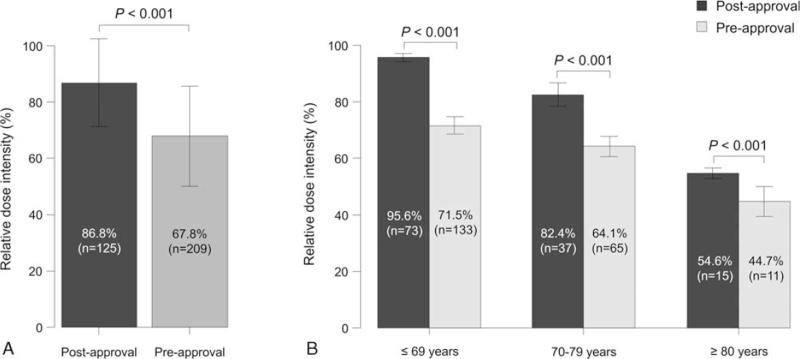
Relative dose intensity in the post and pre-approval groups in the whole study cohort (A) and after stratification by age (B).

RDI was higher in the postapproval group than in the pre-approval group (86.8% vs 67.8%, *P* < .001) (Fig. 2A). Similar results were obtained when the patients were divided into 3 age groups (≤69 years: 95.6% vs 71.5%, *P* < .001; 70–79 years: 82.4% vs 64.1%, *P* < .001; ≥80 years: 54.6% vs 44.7%, *P* < .001) (Fig. 2B). Multivariate analysis revealed that patients with younger age, good PS, and a low Charlson Comorbidity Index, and patients in the postapproval group were significantly associated with RDI ≥85% (age ≥70 years, odds ratio [OR]: 0.09, 95% confidence interval [CI]: 0.04–0.20, *P* < .001; ECOG-PS ≥ 2, OR: 0.33, 95% CI: 0.14–0.81, *P* = .016; Charlson Comorbidity Index ≥ 3, OR: 0.51, 95% CI: 0.27–0.98, *P* = .044; postapproval group, OR: 15.3, 95% CI: 7.45–31.40, *P* < .001) (Table 2).

**Table 2:** Univariate and multivariate analyses of factors associated with RDI ≥ 85%.

	Univariate analysis		Multivariate analysis	
	Odds ratio (95% CI)	*P* value	Odds ratio (95% CI)	*P* value
Age (≥70 yrs)	0.17 (0.09–0.31)	<.001	0.09 (0.04–0.20)	<.001
Sex (male)	0.98 (0.61–1.58)	1		
B-symptoms (+)	0.58 (0.33–1.01)	.043	0.71 (0.36–1.38)	.32
ECOG-PS (≥2)	0.25 (0.11–0.53)	<.001	0.33 (0.14–0.81)	.016
LDH (>ULN)	0.68 (0.42–1.08)	.090		
Ann Arbor stage (3/4)	0.65 (0.41–1.04)	.068		
Extranodal involvement (≥2)	0.62 (0.35–1.07)	.074		
NCCN-IPI (HI/H)	0.27 (0.16–0.44)	<.001	0.77 (0.39–1.51)	.44
CCI (≥3)	0.59 (0.35–0.98)	.033	0.51 (0.27–0.98)	.044
Serum albumin (<3.7 g/dL)	0.52 (0.31–0.85)	.007	1.25 (0.64–2.44)	.52
Initial chemotherapy (postapproval)	6.82 (4.06–11.62)	<.001	15.3 (7.45–31.40)	<.001

CCI = Charlson Comorbidity Index, CI = confidence interval, ECOG-PS = Eastern Cooperative Oncology Group performance status, LDH = lactate dehydrogenase, NCCN-IPI = National Comprehensive Cancer Network-International Prognostic Index, RDI = relative dose intensity, ULN = upper limit of normal.

**Figure 3:**
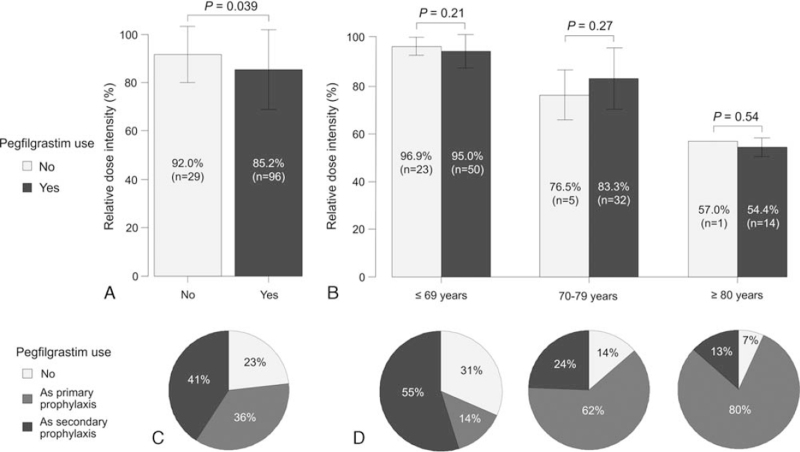
Relative dose intensity in the postapproval group in terms of pegfilgrastim use in the whole cohort (A) and after stratification by age (B). Breakdown of pegfilgrastim use and prophylaxis type in the whole cohort (C) and after stratification by age (D).

Some patients in both groups received a short-acting G-CSF, such as filgrastim or lenograstim, daily for neutropenia treatment. Thirteen patients (6.2%) in the pre-approval group received G-CSF daily. Most of them started receiving a short-acting G-CSF when they became neutropenic while checking their complete blood count in the first course, and during the subsequent courses, planned to receive it for only several days at the timing when neutropenia was anticipated from the first course. In the postapproval group, no patients received short-acting G-CSFs daily as prophylaxis for FN or for RDI maintenance, and 96 patients (76.8%) received pegfilgrastim at least once during R-CHOP. The patients who received pegfilgrastim had a significantly lower RDI than those who did not (85.2% vs 92.0%, *P* = .039) (Fig. 3A). This was considered to be due to the high frequency of elderly patients among those who received pegfilgrastim. In fact, there was no significant difference in the RDI between patients with or without pegfilgrastim when stratified by age group (≥69 years: 95.0% vs 96.9%, *P* = .21; 70–79 years: 83.3% vs 76.5%, *P* = .27; ≥80 years: 54.4% vs 57.0%, *P* = .54) (Fig. 3B). More patients in the older age groups received pegfilgrastim as primary prophylaxis (14%, 62%, and 80% in patients ≤69 years, 70–79 years, and ≥80 years, respectively, *P* < .001) (Fig. 3C, D). Of the patients who did not receive primary prophylaxis using pegfilgrastim, those with poor PS and a low albumin level were more likely to need secondary prophylaxis (Table 3).

**Table 3:** Univariate analysis of factors associated with secondary prophylaxis using pegfilgrastim in the postapproval group.

	Odds ratio (95% CI)	*P* value
Age (>60 yrs)	1.26 (0.45–3.49)	.64
Age (≥65 yrs)	1.16 (0.42–3.26)	.82
Sex (male)	1.16 (0.42–3.20)	.82
B-symptoms (+)	2.58 (0.71–11.93)	.17
ECOG-PS (≥2)	Inf (1.23–Inf)	.023
LDH (>ULN)	1.01 (0.37–2.81)	1
Ann Arbor stage (3/4)	0.61 (0.22–1.67)	.35
Extranodal involvement (≥2)	0.99 (0.32–3.21)	1
NCCN-IPI (HI/H)	1.56 (0.51–5.22)	.45
CCI (≥3)	0.57 (0.20–1.61)	.24
Serum albumin (<3.7 g/dL)	6.62 (1.38–64.10)	.007

CCI = Charlson Comorbidity Index, CI = confidence interval, ECOG-PS = Eastern Cooperative Oncology Group performance status, LDH = lactate dehydrogenase, NCCN-IPI = National Comprehensive Cancer Network-International Prognostic Index, ULN = upper limit of normal.

**Figure 4:**
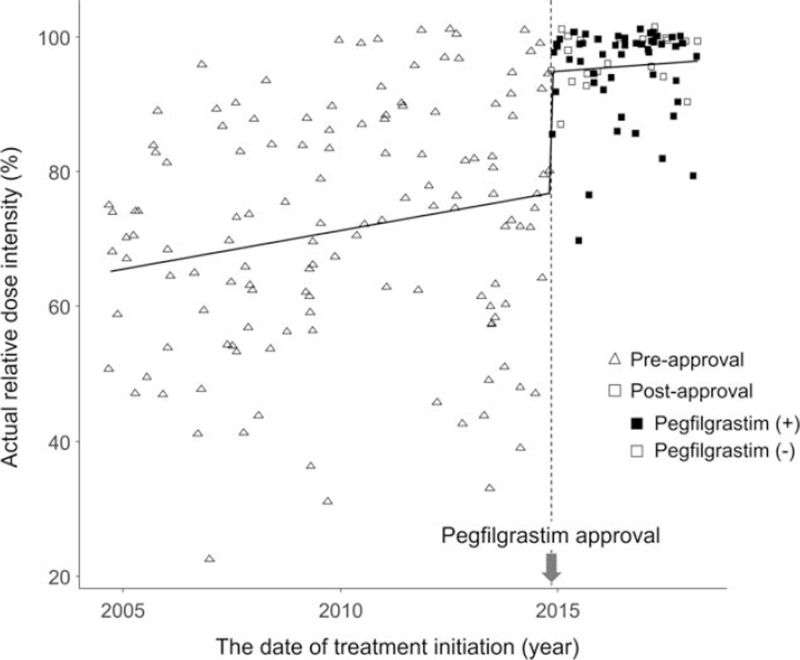
Scatter plot of the date of treatment initiation and relative dose intensity.

Moreover, we estimated the impact of the pegfilgrastim approval on RDI using interrupted time-series analysis. In the analysis of patients aged <70 years, there was a clear rise of RDI at the timing of pegfilgrastim approval (Fig. 4). The change in level of RDI at the pegfilgrastim approval (*β*_*2*_) was shown to be statistically significant (estimated change: 18.1%, *P* < .001) (Table 4).

**Table 4:** Parameter estimates, standard error, and *P* value from the regression model for both the level and trend of RDI before and after the pegfilgrastim approval.

	Estimate	Standard error	t	*P* value
Intercept (baseline level), *β*_*0*_	0.652	0.0256	25.51	<.001
Baseline trend, *β*_*1*_	3.11 × 10^–5^	1.11 × 10^–5^	2.80	.006
Change in level after intervention, *β*_*2*_	0.181	0.0396	4.57	<.001
Change in trend after intervention, *β*_*3*_	−1.88 × 10^–5^	4.80 × 10^–5^	−0.39	.70

RDI = relative dose intensity.

### 3.3 Febrile neutropenia

The incidence of FN was lower in the postapproval group (20% vs 38.3%, *P* < .001). Patients with ECOG-PS ≥2 and those in the postapproval group were significantly associated with FN occurrence, according to multivariate analysis (ECOG-PS ≥2; OR: 2.06, 95% CI: 1.09–3.89, *P* = .027 and postapproval group; OR: 0.45, 95% CI: 0.26–0.76, *P* = .003) (Table 5).

**Table 5:** Univariate and multivariate analysis of factors associated with febrile neutropenia incidence.

	Univariate analysis		Multivariate analysis	
	Odds ratio (95% CI)	*P* value	Odds ratio (95% CI)	*P* value
Age (≥60 yrs)	0.73 (0.43–1.24)	.25		
Sex (male)	1.45 (0.92–2.31)	.19		
B-symptoms (+)	1.37 (0.80–2.34)	.24		
ECOG-PS (≥2)	2.40 (1.32–4.34)	.003	2.06 (1.09–3.89)	.027
LDH (>ULN)	1.57 (0.96–2.60)	.06		
Ann Arbor stage (3/4)	1.77 (1.07–2.96)	.024	1.60 (0.89–2.90)	.12
Extranodal involvement (≥2)	1.21 (0.69–2.07)	.51		
NCCN-IPI (HI/H)	1.61 (0.99–2.65)	.046	0.74 (0.38–1.44)	.37
CCI (≥3)	1.01 (0.60–1.69)	1		
Serum albumin (<3.7 g/dL)	1.90 (1.15–3.12)	.008	1.48 (0.85–2.58)	.17
RDI (≥85%)	0.78 (0.48–1.24)	.067		
Initial chemotherapy (postapproval)	0.40 (0.23–0.69)	<.001	0.45 (0.26–0.76)	.003

CCI = Charlson Comorbidity Index, CI = confidence interval, ECOG-PS = Eastern Cooperative Oncology Group performance status, LDH = lactate dehydrogenase, NCCN-IPI = National Comprehensive Cancer Network-International-Prognostic Index, RDI = relative dose intensity, ULN = upper limit of normal.

### 3.4 Prognosis

**Figure 5:**
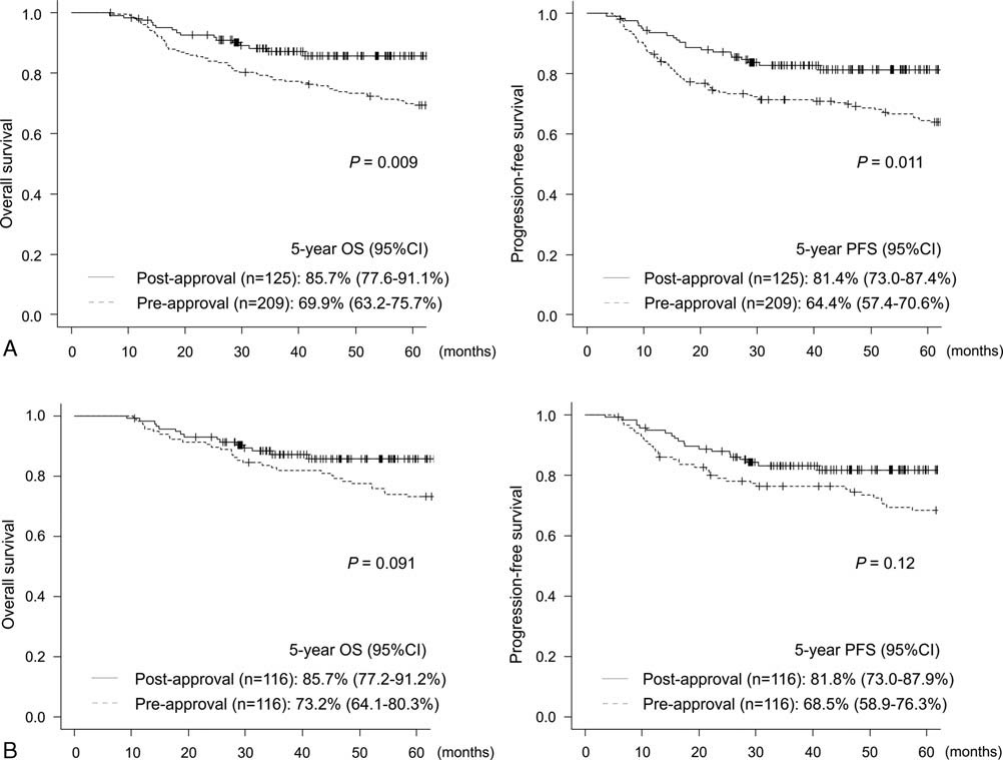
Kaplan-Meier curves of overall survival and progression-free survival in the whole cohort (A) and in the matched-pair analysis matching NCCN-IPI scores (B). NCCN-IPI = National Comprehensive Cancer Network-International Prognostic Index.

**Figure 6:**
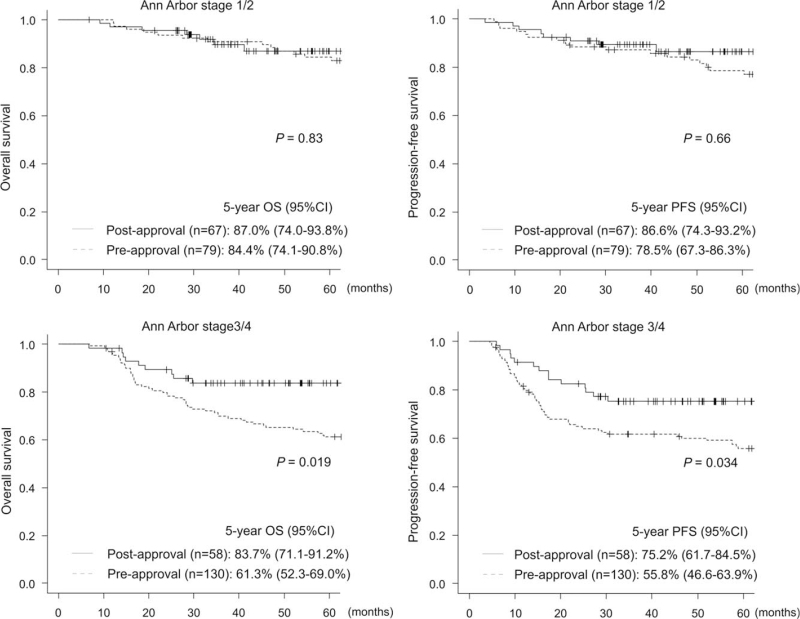
Kaplan-Meier curves of overall survival and progression-free survival stratified by Ann Arbor stage.

**Figure 7:**
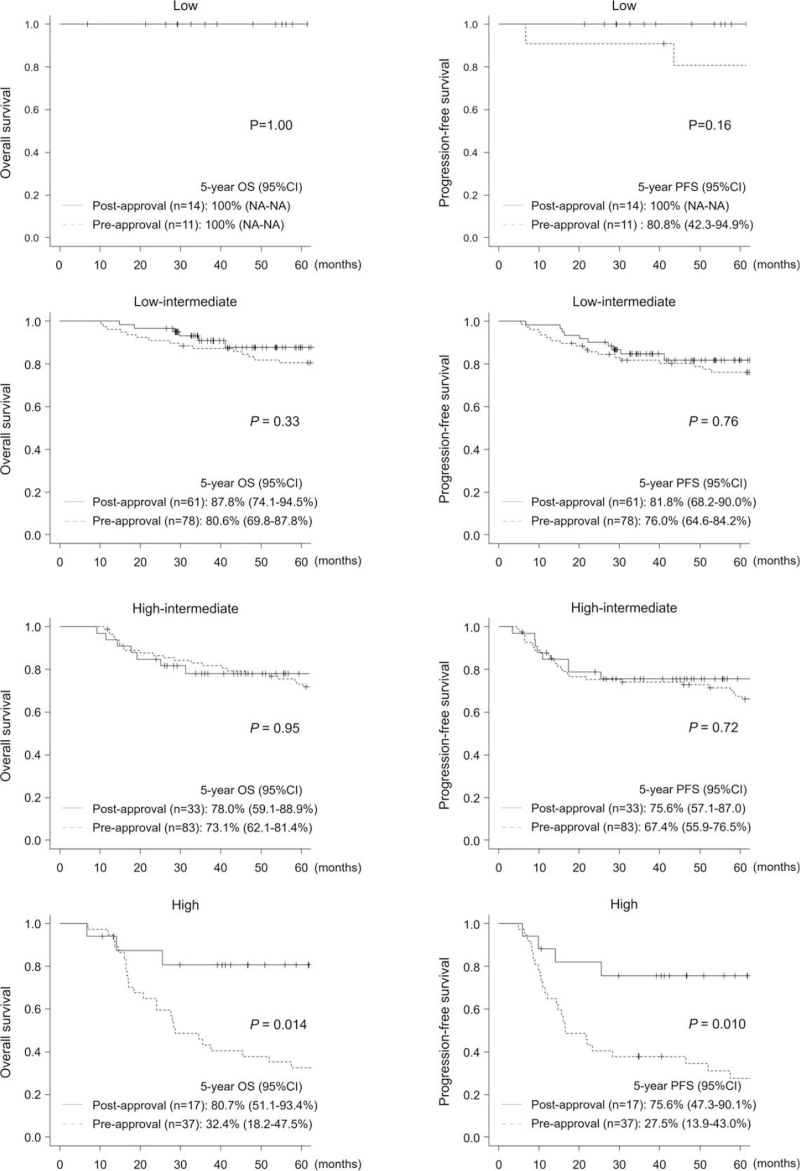
Kaplan-Meier curves of overall survival and progression-free survival stratified by NCCN-IPI. NCCN-IPI = National Comprehensive Cancer Network-International Prognostic Index.

The 5-year OS and PFS were significantly superior in the postapproval group (5-year OS: 85.7% vs 69.9%, respectively, *P* = .009; 5-year PFS: 81.4% vs 64.4%, respectively, *P* = .011) (Fig. 5A). However, the differences were not significant in matched-pair analysis matching NCCN-IPI scores (5-year OS: 85.7% vs 73.2%, respectively, *P* = .091; 5-year PFS: 81.8% vs 68.5%, respectively, *P* = .12) (Fig. 5B). Significant differences between the 2 groups were observed only in the patients with Ann Arbor stage 3/4 (5-year OS: 83.7% vs 61.3%, respectively, *P* = .019; 5-year PFS: 75.2% vs 55.8%, respectively, *P* = .034) (Fig. 6) and among those with a high risk on NCCN-IPI (5-year OS: 80.7% vs 32.4%, respectively, *P* = .014; 5-year PFS: 75.6% vs 27.5%, respectively, *P* = .010) (Fig. 7). No significant differences were observed among the other subgroups. Multivariate analysis for OS showed that a high RDI and low LDH were associated with superior OS (RDI ≥ 85%, hazard ratio: 0.48, 95% CI: 0.27–0.87, *P* = .016; LDH > institutional upper limit of normal, hazard ratio: 2.38, 95% CI: 1.31–4.33, *P* = .005) (Table 6).

**Table 6:** Univariate and multivariate analyses of clinical factors of overall survival.

	Univariate analysis		Multivariate analysis	
	Hazard ratio (95% CI)	*P* value	Hazard ratio (95% CI)	*P* value
Age (>60 yrs)	1.62 (1.06–2.48)	.044	1.57 (0.91–2.68)	.10
Sex (male)	1.35 (0.91–2.02)	.15		
B-symptoms (+)	1.63 (1.03–2.56)	.021	0.99 (0.63–1.55)	.97
ECOG-PS (≥2)	2.49 (1.46–4.26)	<.001	1.45 (0.90–2.34)	.13
LDH (>ULN)	2.93 (1.97–4.37)	<.001	2.38 (1.31–4.33)	.005
Ann Arbor stage (3/4)	2.06 (1.38–3.07)	<.001	1.37 (0.78–2.43)	.28
Extranodal involvement (≥2)	1.99 (1.23–3.22)	<.001	1.17 (0.71–1.90)	.54
NCCN-IPI (IH/H)	2.82 (1.90–4.21)	<.001	0.91 (0.45–1.83)	.79
CCI (≥3)	1.16 (0.76–1.76)	.49		
Serum albumin (<3.7 g/dL)	2.25 (1.48–3.42)	<.001	1.27 (0.79–2.02)	.32
RDI (≥85%)	0.36 (0.23–0.54)	<.001	0.48 (0.27–0.87)	.016
Initial chemotherapy (postapproval)	0.52 (0.33–0.81)	.009	0.72 (0.40–1.29)	.27
Pegfilgrastim administration (+)	1.02 (0.64–1.61)	.94		

CCI = Charlson Comorbidity Index, CI = confidence interval, ECOG-PS = Eastern Cooperative Oncology Group performance status, LDH = lactate dehydrogenase, NCCN-IPI = National Comprehensive Cancer Network-International Prognostic Index, RDI = relative dose intensity, ULN = upper limit of normal.

## 4 Discussion

The present study demonstrated that the RDI of the postapproval group was able to be maintained at a significantly higher level, and that the OS and PFS improved after pegfilgrastim approval in the clinically high-risk groups. To the best of our knowledge, the present report is the first to demonstrate a significant association between pegfilgrastim approval and the prognosis of patients with DLBCL.

Pegfilgrastim, a long-acting G-CSF, was approved for use in Japan in November 2014 to prevent FN induced by chemotherapy. The American Society of Clinical Oncology guidelines clearly state that the reduction of FN is an important clinical outcome.^[16]^ Moreover, FN can lead to infection-related mortality as well as dose reduction during chemotherapy, which in turn can lead to poorer outcomes. Three systematic reviews and meta-analyses demonstrated that the administration of G-CSF, including pegfilgrastim, resulted in better clinical outcomes. In one of these studies, the relative risk of infection-related mortality, early mortality (all-cause mortality during the chemotherapy period), and FN decreased, and that the average RDI significantly increased, in patients receiving G-CSF than in control patients.^[5]^ Another study reported not only a reduction in FN after G-CSF administration, but also the superiority of pegfilgrastim over daily filgrastim, a short-acting G-CSF. Specifically, the FN incidence was significantly lower for pegfilgrastim than filgrastim, with a relative risk of 0.66 (95% CI: 0.44–0.98).^[7]^ Another systematic review demonstrated that primary G-CSF prophylaxis reduced the relative risk of all-cause mortality, particularly in clinical trials with longer follow-up periods where the treatment was for curative intent and survival was the primary outcome.^[8]^

In terms of the relationship between malignant lymphoma and G-CSF, a meta-analysis of 13 randomized control studies for malignant lymphoma concluded that G-CSF/GM-CSF prophylaxis significantly reduced the incidence of FN, neutropenia, and infection but did not significantly improve freedom from treatment failure or OS.^[17]^ A randomized prospective trial reported that primary prophylaxis with pegfilgrastim reduced FN incidence and hospitalizations resulting from neutropenia or FN in patients with NHL aged 65 years or older.^[18]^

The major guidelines recommend primary G-CSF prophylaxis for patients with a high FN risk (≥20%) receiving chemotherapy and patients classified as intermediate risk (10%–19%) with risk factors of FN, such as older age, bone marrow invasion, poor PS, malnutrition, etc.^[16,19,20]^ The FN incidence in patients with DLBCL receiving R-CHOP is reportedly 18% to 19%, which is considered to be intermediate-risk.^[20]^ Therefore, administration of pegfilgrastim as primary prophylaxis is recommended from the first R-CHOP cycle in patients with risk factors of FN. In the present study, pegfilgrastim was more often administered as a secondary prophylaxis to patients with poor PS and a low albumin level. Furthermore, it was also shown that the incidence of FN was higher in patients with a low albumin level. Based on these findings, primary prophylaxis with pegfilgrastim may be considered as a viable option for these patients.

As far as could be ascertained, no clear evidence indicates that the introduction of pegfilgrastim into DLBCL treatment has improved patients’ prognosis. To investigate the impact of pegfilgrastim, the present study compared clinical outcomes before and after approval of the drug. Our study found that the FN incidence decreased while the RDI of R-CHOP increased, as previously reported, and that the OS and PFS significantly improved after pegfilgrastim approval in high-risk groups. Moreover, multivariate analysis of OS found that high RDI led to improved prognosis, in line with previous reports.^[1,2,21]^

Interrupted time-series analysis is a quasi-experimental method of statistical analysis involving tracking a long-term period before and after a point of intervention to assess the intervention's effects. In this analysis, data are collected at multiple time points both before and after an interruption. Modeling of the data in the pre-interruption period allows estimation of the underlying secular trend, which when modeled correctly and extrapolated into the postinterruption time period, yields a counterfactual for what would have occurred in the absence of the interruption. In this way, differences between the counterfactual and observed data at various points of postinterruption can be estimated.^[22]^ The key assumptions we have to make are that the characteristics of the populations remain unchanged throughout the study period and that there is no comparator against which to adjust the results for changes that should not be attributed to the intervention itself.^[23]^ Because there were unlikely to be any other changes that could influence the RDI around November 2014 (the approval of pegfilgrastim), we considered that this method could be applied in this study. Actually, the analysis demonstrated the evident “jump” of RDI after the pegfilgrastim approval, suggesting that pegfilgrastim approval contributed to the improvement of RDI. Of note, there was no significant difference in the RDI between patients with or without pegfilgrastim administration (Fig. 3B). Pegfilgrastim was considered to contribute to maintaining a high RDI in patients whose RDI were likely to be low before pegfilgrastim became available, leading to the high level of RDI in the entire postapproval group.

There were fewer patients with advanced stage DLBCL or a high-intermediate or high risk on the NCCN-IPI in the postapproval group, which could lead to the difficulty of accurately estimating the impact of pegfilgrastim approval on survival outcomes. In fact, matched-pair analysis matching NCCN-IPI scores showed no significant differences in OS or PFS, even though the analysis without matching demonstrated significant differences. However, it was more noteworthy that improved OS and PFS in the postapproval group were observed in the advanced stage and NCCN-IPI high-risk groups. Considering together that pegfilgrastim approval was not a significant prognostic factor on multivariate analysis (Table 6), the lack of a significant differences in the results in the entire study population was acceptable. Instead, these results stressed that maintaining a high RDI was important to achieve good survival outcomes in the clinically high-risk groups. In addition, it might be possible to reduce the intensity of R-CHOP in the clinically low-risk groups. In fact, some recent studies reported that 4 cycles of R-CHOP were sufficient for patients with localized or low-risk DLBCL.^[24,25]^

This study has some limitations. First, it was a retrospective study, making it impossible to assess all the factors that might have influenced the clinical outcomes. Especially, evaluating the impact of pegfilgrastim could be difficult if there were a substantial number of patients receiving short-acting G-CSFs daily for FN prophylaxis or RDI maintenance. However, such patients comprised only 6.2% of the pre-approval group and they received these drugs only for a short term. Although prophylactic G-CSFs administration was considered for patients with indications similarly before pegfilgrastim was approved, most patients did not receive them due to the heavy burden of daily hospital visits but were treated by reducing the dose of chemotherapeutic drugs and/or extending the administration interval if necessary. Furthermore, the standard therapeutic strategy for DLBCL has not changed for more than 15 years. Based on these facts, it is likely that the pre-approval group served as a good historical control for investigating the impact of pegfilgrastim approval on clinical outcomes in patients with DLBCL. Second, patients who did not complete 6 cycles of R-CHOP were excluded. Because these patients were considered to have a poor prognosis, their exclusion might also have impacted the results. For reference, the treatment completion rate and clinical outcomes of all patients with DLBCL receiving R-CHOP improved after pegfilgrastim approval (treatment completion rate: 78.5% vs 65.9%, *P* = .003; 5-year OS: 82.3% vs 61.7%, *P* < .001; 5-year PFS: 73.0% vs 56.0%, *P* = .007, in the postapproval and pre-approval groups, respectively). Despite these limitations, the present study is the first to demonstrate that pegfilgrastim has the potential to contribute to improving survival outcomes in patients with DLBCL by maintaining high RDI. Considering that we included consecutive patients who received R-CHOP for curative intent, the external validity of the impact of pegfilgrastim was appeared to be high.

## 5 Conclusion

After pegfilgrastim approval, the RDI of R-CHOP was able to be maintained at higher levels, the incidence of FN was lower, and significantly better clinical outcomes were achieved in clinically high-risk groups, suggesting that maintaining a high RDI in R-CHOP by administering pegfilgrastim to those who need it to keep the RDI at a high level is important for achieving favorable outcomes in patients with DLBCL.

## Acknowledgments

The authors would like to thank the nursing staff at Tokyo Metropolitan Cancer and Infectious Diseases Center, Komagome Hospital for their excellent patient care.

## Author contributions

**Conceptualization:** Tatsu Shimoyama.

**Investigation:** Yuka Morita, Yusuke Kanemasa, Yuki Sasaki, An Ohigashi, Taichi Tamura, Shohei Nakamura, Yu Yagi, Akihiko Kageyama, Yasushi Omuro, Tatsu Shimoyama.

**Writing – original draft:** Yuka Morita.

**Writing – review & editing:** Yusuke Kanemasa.

## References

[1] Terada Y Nakamae H Aimoto R. Impact of relative dose intensity (RDI) in CHOP combined with rituximab (R-CHOP) on survival in diffuse large B-cell lymphoma. J Exp Clin Cancer Res 2009;28:116. 19689822 10.1186/1756-9966-28-116 PMC2743657

[2] Hirakawa T Yamaguchi H Yokose N Gomi S Inokuchi K Dan K. Importance of maintaining the relative dose intensity of CHOP-like regimens combined with rituximab in patients with diffuse large B-cell lymphoma. Ann Hematol 2010;89:897–904. 20414658 10.1007/s00277-010-0956-7

[3] Gutiérrez A Bento L Bautista-Gili AM. Differential impact of relative dose-intensity reductions in diffuse large B-cell lymphoma treated with R-CHOP21 or R-CHOP14. PLoS One 2015;10:e0123978. 25909361 10.1371/journal.pone.0123978 PMC4409365

[4] Friedberg JW. Relapsed/refractory diffuse large B-cell lymphoma. Hematology Am Soc Hematol Educ Program 2011;2011:498–505. 22160081 10.1182/asheducation-2011.1.498

[5] Kuderer NM Dale DC Crawford J Lyman GH. Impact of primary prophylaxis with granulocyte colony-stimulating factor on febrile neutropenia and mortality in adult cancer patients receiving chemotherapy: a systematic review. J Clin Oncol 2007;25:3158–67. 17634496 10.1200/JCO.2006.08.8823

[6] Pinto L Liu Z Doan Q Bernal M Dubois R Lyman G. Comparison of pegfilgrastim with filgrastim on febrile neutropenia, grade IV neutropenia and bone pain: a meta-analysis of randomized controlled trials. Curr Med Res Opin 2007;23:2283–95. 17697451 10.1185/030079907X219599

[7] Cooper KL Madan J Whyte S Stevenson MD Akehurst RL. Granulocyte colony-stimulating factors for febrile neutropenia prophylaxis following chemotherapy: systematic review and meta-analysis. BMC Cancer 2011;11:404. 21943360 10.1186/1471-2407-11-404 PMC3203098

[8] Lyman GH Dale DC Culakova E. The impact of the granulocyte colony-stimulating factor on chemotherapy dose intensity and cancer survival: a systematic review and meta-analysis of randomized controlled trials. Ann Oncol 2013;24:2475–84. 23788754 10.1093/annonc/mdt226 PMC3841419

[9] Swerdlow SH, Campo E, Harris NL, et al. WHO Classification of Tumours of Haematopoietic and Lymphoid Tissues. 4th ed. World Health Organization Classification of Tumours. International Agency for Research on Cancer; 2008:439 p.

[10] Swerdlow SH, Campo E, Harris NL, et al. WHO Classification of Tumours of Haematopoietic and Lymphoid Tissues. Rev. 4th ed. World Health Organization Classification of Tumours. International Agency for Research on Cancer; 2017:585 p.

[11] Zhou Z Sehn LH Rademaker AW. An enhanced International Prognostic Index (NCCN-IPI) for patients with diffuse large B-cell lymphoma treated in the rituximab era. Blood 2014;123:837–42. 24264230 10.1182/blood-2013-09-524108 PMC5527396

[12] Mori M Niitsu N Takagi T. Reduced-dose chop therapy for elderly patients with non-Hodgkin's lymphoma. Leuk Lymphoma 2001;41:359–66. 11378549 10.3109/10428190109057991

[13] Bernal JL Cummins S Gasparrini A. Interrupted time series regression for the evaluation of public health interventions: a tutorial. Int J Epidemiol 2017;46:348–55. 27283160 10.1093/ije/dyw098 PMC5407170

[14] Ramsay CR Matowe L Grilli R Grimshaw JM Thomas RE. Interrupted time series designs in health technology assessment: lessons from two systematic reviews of behavior change strategies. Int J Technol Assess Health Care 2003;19:613–23. 15095767 10.1017/s0266462303000576

[15] Huitema BE McKean JW. Identifying autocorrelation generated by various error processes in interrupted time-series regression designs: a comparison of AR1 and portmanteau tests. Educ Psychol Meas 2007;67:447–59.

[16] Smith TJ Bohlke K Lyman GH. Recommendations for the use of WBC growth factors: American Society of Clinical Oncology clinical practice guideline update. J Clin Oncol 2015;33:3199–212. 26169616 10.1200/JCO.2015.62.3488

[17] Bohlius J Herbst C Reiser M Schwarzer G Engert A. Granulopoiesis-stimulating factors to prevent adverse effects in the treatment of malignant lymphoma. Cochrane Database Syst Rev 2008;2008:CD003189. 10.1002/14651858.CD003189.pub4 PMC7144686 18843642

[18] Balducci L Al-Halawani H Charu V. Elderly cancer patients receiving chemotherapy benefit from first-cycle pegfilgrastim. Oncologist 2007;12:1416–24. 18165618 10.1634/theoncologist.12-12-1416

[19] Aapro MS Bohlius J Cameron DA. 2010 update of EORTC guidelines for the use of granulocyte-colony stimulating factor to reduce the incidence of chemotherapy-induced febrile neutropenia in adult patients with lymphoproliferative disorders and solid tumours. Eur J Cancer 2011;47:08–32. 10.1016/j.ejca.2010.10.013 21095116

[20] Becker PS Griffiths EA Alwan LM. NCCN guidelines insights: hematopoietic growth factors. J Natl Compr Canc Netw 2020;18:12–22. Version 1.2020. 31910384 10.6004/jnccn.2020.0002

[21] Lyman GH Dale DC Friedberg J Crawford J Fisher RI. Incidence and predictors of low chemotherapy dose-intensity in aggressive non-Hodgkin's lymphoma: a nationwide study. J Clin Oncol 2004;22:4302–11. 15381684 10.1200/JCO.2004.03.213

[22] Zhang F Wagner AK Soumerai SB Ross-Degnan D. Methods for estimating confidence intervals in interrupted time series analyses of health interventions. J Clin Epidemiol 2009;62:143–8. 19010644 10.1016/j.jclinepi.2008.08.007 PMC3828652

[23] Kontopantelis E Doran T Springate DA Buchan I Reeves D. Regression based quasi-experimental approach when randomisation is not an option: interrupted time series analysis. BMJ 2015;350:h2750. 26058820 10.1136/bmj.h2750 PMC4460815

[24] Poeschel V Held G Ziepert M. Four versus six cycles of CHOP chemotherapy in combination with six applications of rituximab in patients with aggressive B-cell lymphoma with favourable prognosis (FLYER): a randomised, phase 3, non-inferiority trial. Lancet 2019;394:2271–81. 31868632 10.1016/S0140-6736(19)33008-9

[25] Persky DO Li H Stephens DM. Positron emission tomography-directed therapy for patients with limited-stage diffuse large B-cell lymphoma: results of Intergroup National Clinical Trials Network Study S1001. J Clin Oncol 2020;38:3003–11. 32658627 10.1200/JCO.20.00999 PMC7479758

